# Computer model of a spectrum analyzer for a virtual laboratory: development and introduction to the educational process

**DOI:** 10.7717/peerj-cs.1130

**Published:** 2022-11-03

**Authors:** Dmitry Gubsky, Yevgeniya Daineko, Madina Ipalakova, Anatoly Kleschenkov, Dana Tsoy

**Affiliations:** 1 Mixed Reality Lab, International Information Technology University, Almaty, Kazakhstan; 2 The Department of Radio Engineering, Electronics and Telecommunications, International Information Technology University, Almaty, Kazakhstan; 3 The Department of Computer Engineering, International Information Technology University, Almaty, Kazakhstan

**Keywords:** UHF devices, Microwaves, Distance education, Radiophysics, Virtual laboratories

## Abstract

The worldwide COVID-19 pandemic has changed the development plans of every country. Instead, governments had to constantly deal with ever-emerging issues in healthcare, education, economics and industry. As a result, there has been an accelerated introduction of digitalization in these spheres. Thus, an increasing number of people have started using electronic services that have improved their digital literacy. This feature has had a positive impact on society and helped to create new interaction tools between populations and governments, students and institutions, customers and companies. The article aims to analyze how studying radio electronics can be improved by involving new tools and how they can be applied in distance learning. This work presents the results of the development and application of a virtual radio signal simulation in the educational process in the form of a laboratory practicum. Working on this approach required specific research in the field; the foreign experience was observed and studied. The review allowed us to find out how digitalization and the application of digital tools affect the behaviour, cognition, and overall performance of students during the pandemic. The authors conducted a questionnaire among students to evaluate the features of the virtual laboratory work and their effect on the educational process. The results analyzed are given in the article. They showed that students highly appreciate the introduction of such tools in learning. Moreover, like the entire laboratory, the proposed model can be used in the educational process offline and with distance learning. Finally, the article describes the experience and results of the software package’s development and integration for the spectrum analyzer’s computer model and virtual laboratory work using the MS VS environment in C ++. The results of the conducted work demonstrate the versatility of the proposed approach, its positive impact on the educational process, high potential in the other spheres.

## Introduction

Laboratory work deserves special attention when teaching students of technical majors, among all the activity types. In this case, modern measuring equipment must be affordable; however, its high price limits its application in the educational process. On the other hand, distance learning has become widespread in recent years. One of the reasons for this was the COVID-19 pandemic when most educational institutions were forced to switch to online learning.

One of the methods to implement distance learning for technical specialties is to use virtual labs and setups. Therefore, special virtual workshops and stand-alone computer models of measuring instruments and devices are required. Their creation is a relevant task, especially in Kazakhstan where institutions are not equally equipped with necessary devices. It badly affects students’ learning outcomes as they do not get enough level of knowledge about the processes and devices being studied.

Thus, the COVID-19 pandemic has accelerated the transition to distance learning in Kazakhstan though the infrastructure was not prepared for this. For instance, there was no well-established communication system online between students, teachers, and university management. However, despite this, according to Seilkhan et al. (2022) the main problem was a lack of practical knowledge for students of technical specialties.

These issues led to the development of many virtual labs, including in the natural and technical sciences (Daineko, Dmitriyev & Ipalakova, 2017). Virtual work using various graphical environments for process modelling allows students to fully master the material being studied and consolidate their knowledge in practice.

Virtual laboratory work in the field of radio physics and radio communication consists of several areas, such as the development of virtual measuring instruments; signal processing and the study of modulation types; research of wireless networks; design of antenna systems; electromagnetic compatibility of radio electronic devices and electromagnetism; study of transmission lines and devices in the VHF and UHF bands, as well as virtual work in the field of electronics. Therefore, in creating virtual labs, two directions can be distinguished: developing models of various devices that correctly describe the studied physical processes and the development of virtual measuring instruments.

### Literature review

COVID-19 caused many problems and revealed the weaknesses of each country. Apart from medicine, the most critical and complex transition relates to education. There were about 1.6 billion students affected by the pandemic in more than 200 countries (Mohammed, 2022). Almost all institutions and educational organizations switched to distance learning because of the epidemiologic conditions. It led not only to educational organization troubles but also to social ones. Karakose, Yirci & Papadakis (2021) discovered that unprepared systems affected relations in families and within school administrations. It demonstrated the importance of preparedness of the state systems to react fast and effectively under critical circumstances.

Thus, for the development of the virtual content of the courses in the field of radio engineering, such as labs, simulation models, and others the researchers use various approaches. In the work (Manukyan, 2021) authors describe their user experience with the software that makes studying of radio engineering and electronics easier through rich visualization. They introduced TAC, AutoDesk Circuits, and Arduino library into the educational process and concluded that software of this type can be adjusted to different types of programs, thanks to the modular system. This means that it provides users with many devices and equipment types that later can be used in different setups.

Alpotte et al. (2017) present virtual tools and 3D models to get acquainted with the concepts of wireless sensor networks (WSN). The authors use the eWISENS simulation tool and multilingual learning environment. The article (Losilla, 2017) describes the NetEval learning tool. It aims to design and evaluate heterogeneous networks consisting of wired and wireless technologies and allows for remote use.

To study wireless networks using Wi-Fi technology, an educational and real-time research platform, RTWiFi-Lab, was developed (Küçük, 2018). It consists of seven experiments aimed at studying wireless and digital communications, such as signal detection, signal modulation, demodulation, and the study of OFDM (orthogonal frequency-division multiplexing) technology.

Vasilenko & Zhabin (2020) describe the experience of LabVIEW during a radio engineering course. They developed the course based on the features of LabVIEW and using additional extensions such as the NI ELVISmx kit. It supplied students with different laboratory tasks on each of the topics of the course.

The authors (Juan-Llacer et al., 2019) presented a virtual tutorial on calculating electronic coverage. It contains various practical exercises that enable students to acquire knowledge about radio communication system planning, radio link budgeting, and network coverage.

In Juan-Llacer et al. (2019) and Singh et al. (2019), the authors present an augmented reality learning environment in the field of electronics. Thus, the authors propose a system that automatically identifies an integrated circuit using a smartphone and a breadboard (Avilés-Cruz & Villegas-Cortez, 2019), and in the article (Singh et al., 2021) the authors developed virtual 3D models. Interaction with the 3D models of laboratory equipment in an augmented reality environment causes additional interest in students to study the material and perform labs.

Coruk, Yalcinkaya & Kara (2020) presents the development of training material for the course of communication systems. During the lab implementation, students get acquainted with the main parameters of antennas, as well as with their several types (antenna arrays, Hertz dipole).

Despite the abundance of projects, not all of them created models of devices with a realistic interface (Gubsky et al., 2021; Singh et al., 2021; Titov et al., 2016). Thus, the authors developed the virtual 3D models of the oscilloscope and signal generator, and a realistic photo interface was implemented. Various modeling packages (*e.g.*, Maple, LabView, MATLAB, Simulink), programming languages, and technologies are ready for their application to develop virtual labs.

The aim of the examined project (Volovyk et al., 2021) is to study the physics of the observed processes and master students’ methods of working with modern measuring equipment.

Thus, the analysis of these papers, projects, and reports at conferences showed that creating virtual labs is a relevant task. The simulated instruments and devices must look like the actual ones and provide access to the real controls. The proposed approach to creating and applying computer models of measuring instruments and studied devices allows for the development of easily configurable and expandable virtual laboratories designed to study UHF devices and, most importantly, gain skills in working with measuring equipment.

In addition, the analysis of existing developments showed that, at present, a substantial number of works are about the modeling of radio physics problems. However, the update of labs is complicated, and the simple inclusion of the created models of devices under test (DUT) in other works is almost impossible. It is also worth noting that the user interface of most computer models of the studied devices and measuring instruments have only a conceptual similarity with the actual equipment or does not have it at all. Therefore, such models cannot give practical skills in working with them; however, this feature is essential for a distance learning model.

Therefore, the further development of the previously created open virtual laboratory (Gubsky et al., 2021) for the study of UHF devices, and the updating of its functionality by introducing new measuring devices, such as a spectrum analyzer, is simply necessary and relevant. This expands its capabilities.

The novelty of this work lies in the formulation and solution of two interrelated problems. One of them is the creation of a spectrum analyzer model and laboratory work on the study of the summation of radio signals. The other, not less important, is the introduction of the developed virtual laboratory work into the educational process and the study of its impact on the quality of the educational process, and the analysis of the obtained results. The logic implementation is highlighted in detail in the section “An example of a virtual lab implementation”. Particular attention is paid to the interaction of various devices, the exchange of data between them, and the order of processing and conversion of radio signals. Along with this the results of the practical use of the labs are shown and analyzed in the form of the survey in the section “Application in the educational process”. The article describes a model of a spectrum analyzer and a model of a ring power adder used as devices being studied to obtain primary skills in making measurements using a spectrum analyzer.

### Problem statement

Spring 2020 forced digitalization of the all-important processes and at the same time demonstrated the weaknesses of each system and made problematic areas more complex. For instance, Kazakhstani state systems were not prepared for such a massive connection to the newly created infrastructures. Education, as one of the most critical and risky areas, according to Reimers (2022), as well as students, teachers, and staff of the institutions experienced a strong impact. The pandemic highlighted many problems in education in the country (Seilkhan et al., 2022), including poor Internet connection, lack of equipment, experience, methodology, and ways of communication between teachers and students. The solution that can solve most of these problems is a common electronic platform with virtual applications that can replace actual equipment. In this article, we propose a computer simulation used in a virtual lab. Thus, students get the experience close to the real one and are not limited with time, area, or necessary equipment. The evaluation of the suggested approach and its effect on the educational process is also presented.

The development of a computer model of a spectrum analyzer should be carried out considering the previously developed requirements (Gubsky & Zemlyakov, 2019) for models of measuring instruments to include in the virtual laboratory. The fulfillment of these requirements makes it possible to create a new class of devices designed to study the spectrum of radio signals. Their integration into the virtual laboratory significantly expands its functionality.

In this case, the interface of the computer model of the spectrum analyzer must fully mimic the actual instrument. It is also necessary to implement functionality that allows a user to carry out all the necessary measurements and use most of the capabilities of the measuring device to study the spectrum of the input signal.

To demonstrate the operation of the spectrum analyzer, a DUT model (virtual laboratory layout) is developed that combines two high-frequency signals and feeds them to the spectrum analyzer’s input. The created device also meets all previously formulated requirements and is a part of the virtual laboratory.

### Spectrum analyzer simulation

Let us consider the process of creating a computer model of a spectrum analyzer on the example of a device from Rohde & Schwarz (Munich, Germany) of the Hameg series (HMS 3000). This device has rich functionality, but usually, a user needs a small number of essential functions to develop necessary maintenance. These include setting the sweep frequencies (FREQ menu), span and center frequency (SPAN menu); amplitude parameters (AMPL menu), and for the convenience of measurements—setting up the measuring line (LINES menu); work with markers (MARKER menu) as well as with the data input block (block of DATA buttons). The current computer model of the spectrum analyzer has all these functions. Therefore, the computer interface of the model fully matches the actual device. The result corresponds to the previously developed concept of the creation of virtual measuring instruments.

**Figure 1:**
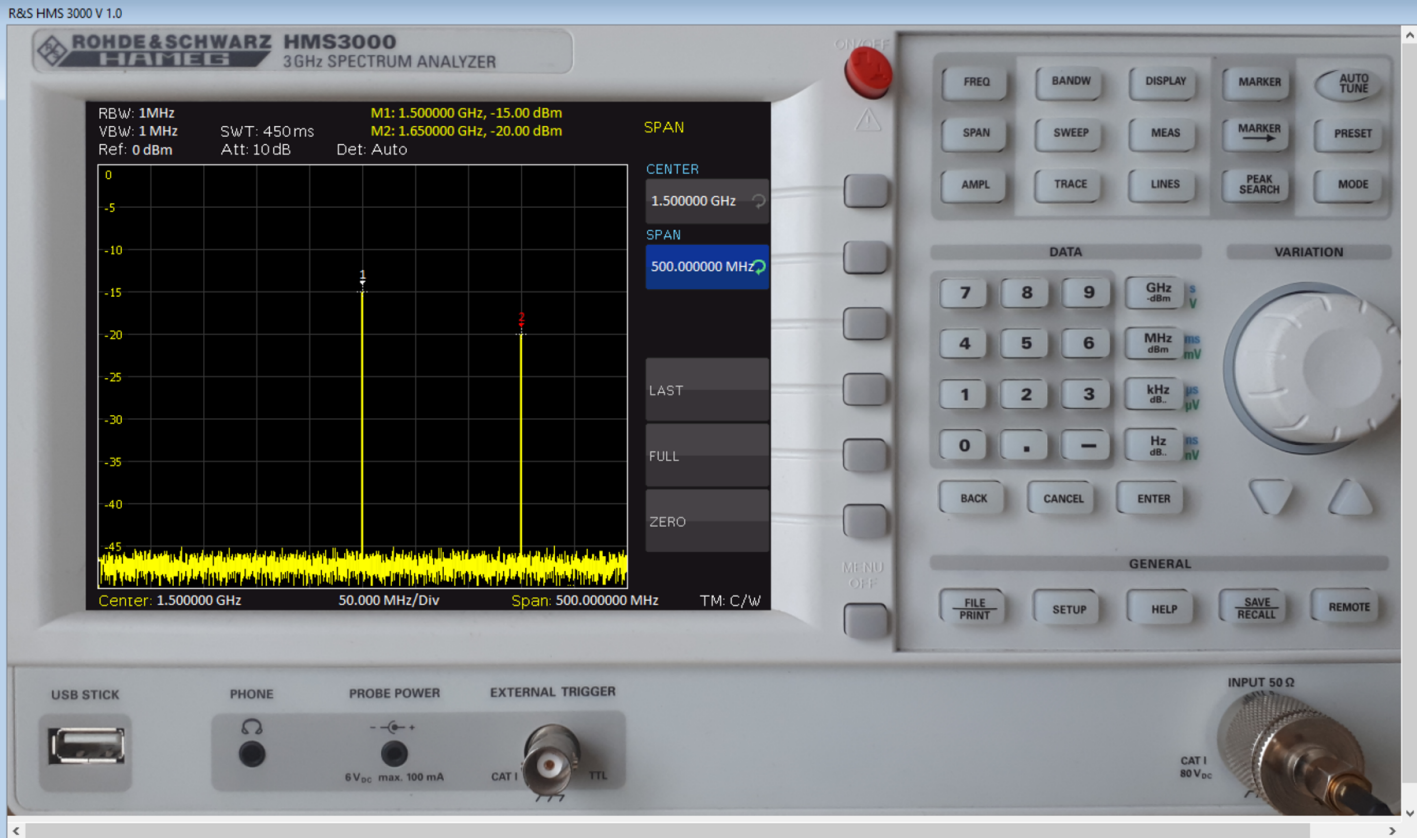
Computer interface of the model of the spectrum analyzer.

Furthermore, the computer model’s interface completely repeats the interface of the equipment, based on the photographs of the actual device. The commands are called by clicking a computer mouse. The program defines which button of the device is pressed, and the corresponding menu appears on the screen of the model, or the corresponding handler performs specific actions, including data entry using the DATA button. All this makes the created model look real and creates the impression of working with an actual measuring device. Figure 1 shows the computer interface of the created spectrum analyzer model.

It should be noted that the spectrum analyzer has only an input to which the radio signal being studied by the user is fed in time representation. Processing it, the spectrum analyzer performs a Fourier transform. After that, if the input signal does not change, the spectrum analyzer works autonomously. Its work comes down to processing the previously obtained spectral representation of the radio signal.

However, to provide the ability to connect various devices (sources of different radio signals) to the spectrum analyzer model, the Fourier transform was moved outside the created model. On the one hand, this can be considered a disadvantage, however, it made it possible to expand the scope of the created model since the type of a specific input is not determined. In this formulation, we need the spectrum analyzer model to receive an array describing the spectral components of the input radio signal. In our case, this is the frequency of the spectral component and its amplitude. Suppose this data array is transferred to the spectrum analyzer. In that case, it can further be considered completely autonomous, and its functionality is only responsible for displaying this array on the screen following the user’s settings and outputting markers and a measuring line. But when the input signal changes, the spectrum analyzer must receive a new (recalculated) array of spectral components.

This approach slightly changed the actual order of signal processing since the Fourier transform is performed outside the spectrum analyzer. But this change gives us versatility since any radio signal processed and presented as an array of spectral components can be studied using this spectrum analyzer model.

Thus, the connected device must output the signals as a discrete array of pairs of values (signal frequency and amplitude). This array is obtained by processing and Fourier transforming the response of the device being studied to input actions. The array’s length depends on the number of spectral components representing the studied signal.

In this case, our task is reduced to transferring the model of the measuring device to the spectral representation of the studied radio signal. Then the spectrum analyzer model displays it on its screen under the operating modes set by the user.

At the same time, when the user presses the corresponding button of the device, the data is processed and redrawn on the screen of the device model. So, for example, when the LINES menu is called by the button pressed on the device, the entry of the UPPER POS value becomes available. For this, the functionality of processing the pressing of the buttons of the DATA block by the user is implemented. Upon the input completion (for example, pressing the ENTER button), the entered data is processed, and the measuring line is redrawn. Setting markers work similarly (MARKER menu). However, a set of functions for searching and moving along the extrema of the spectrum of the displayed radio signal has a more complex implementation. Thus, when receiving data from the device being studied (the radio signal being studied), a special array of extrema of spectral values is created, which is used to search when calling the corresponding functions. For example, calling the PEAK function of the MARKER menu searches for the maximum value of the spectral component (its frequency and amplitude), and after that, the marker drawing block is called, and its values are displayed on the screen of the measuring instrument model.

The data on the screen is updated based on the user’s input. For example, the user changes the settings of the device, and then the spectrum span refreshes according to them. The signals from other devices can also affect the displayed data. In this case, the spectrum analyzer model must receive a new set of spectral components, which are processed and displayed on the instrument screen.

This mechanism is implemented using the concept of connecting devices in the configurator (the output of the device being studied is connected to the input of the spectrum analyzer) and exchanging data between them by calling the appropriate functions through the signal/slot mechanism, which is defined in the AbstractFunc abstract class. The virtual function pr_func() is redefined in the developed software of the spectrum analyzer, the parameters of which and their descriptions are given in Table 1.

**Figure 2:**
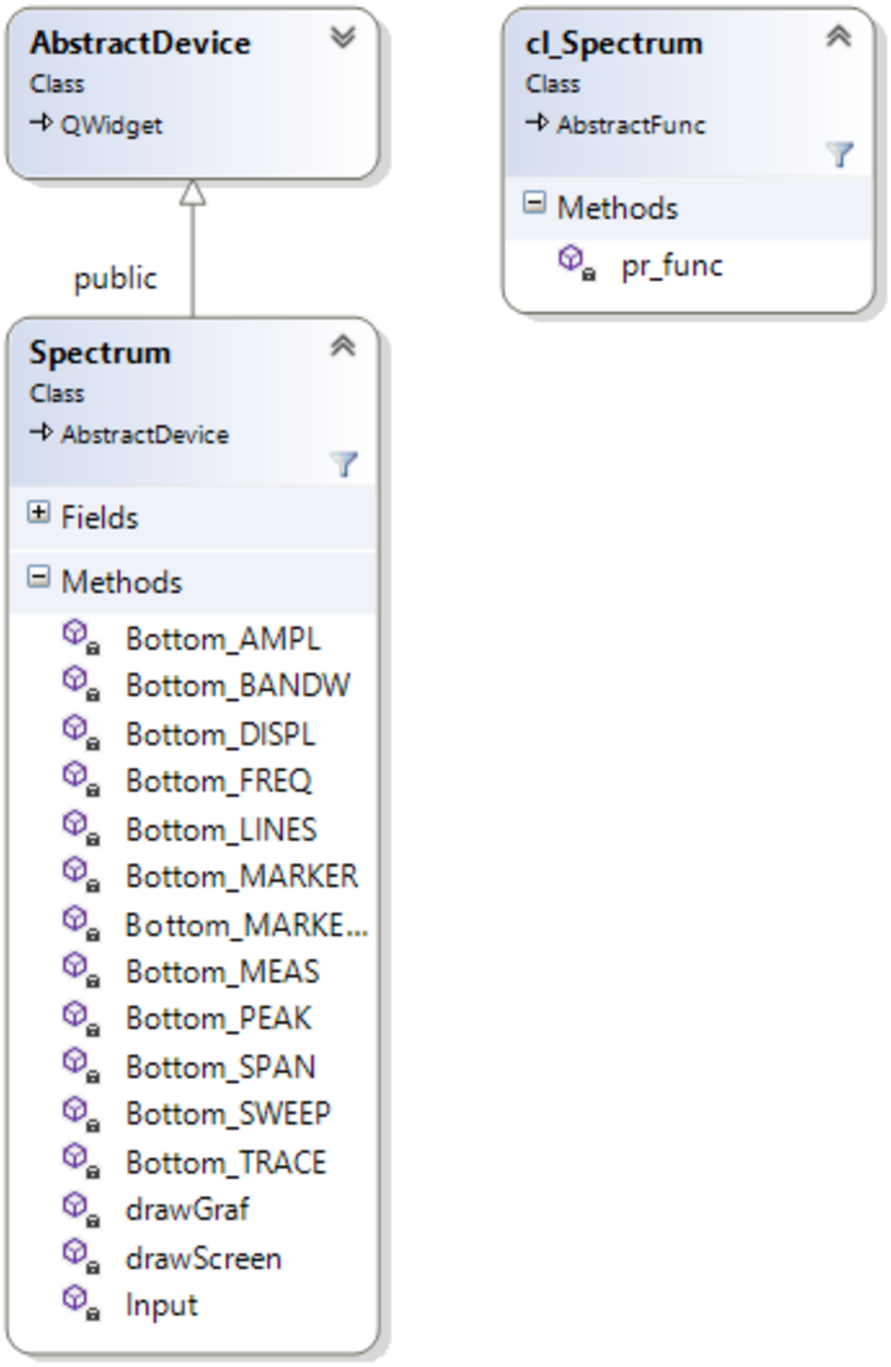
Class diagram.

The main methods of the developed Spectrum class are shown in the class diagram (Fig. 2). Thus, the Bottom_ methods are responsible for implementing the functionality of the menu buttons, and drawGraf and drawScreen are responsible for displaying all information about the device model on the screen, considering the user’s settings. Figure 2 shows that the Spectrum class is a descendant of the AbstractDevice class. This allowed the created device model (after compiling the software, the dll file) to be included in the virtual laboratory and ensured the operation of the previously developed mechanism for data exchange between the measuring instrument and the device being studied.

**Table 1:** Examples of calling the data exchange function.

Set of parameters	Description
*n*, 0, 0	*n*—the size of the arrays (the number of spectral components in the input signal)
	*Used to create dynamic arrays that store information about the spectral representation of the input radio signal*
*i*, *f*, *a*	Transmission of data on the *i*-th component of the spectrum:
	*f*—spectrum component frequency (MHz)
	*a*—amplitude of a given spectrum component (dBm)

**Figure 3:**
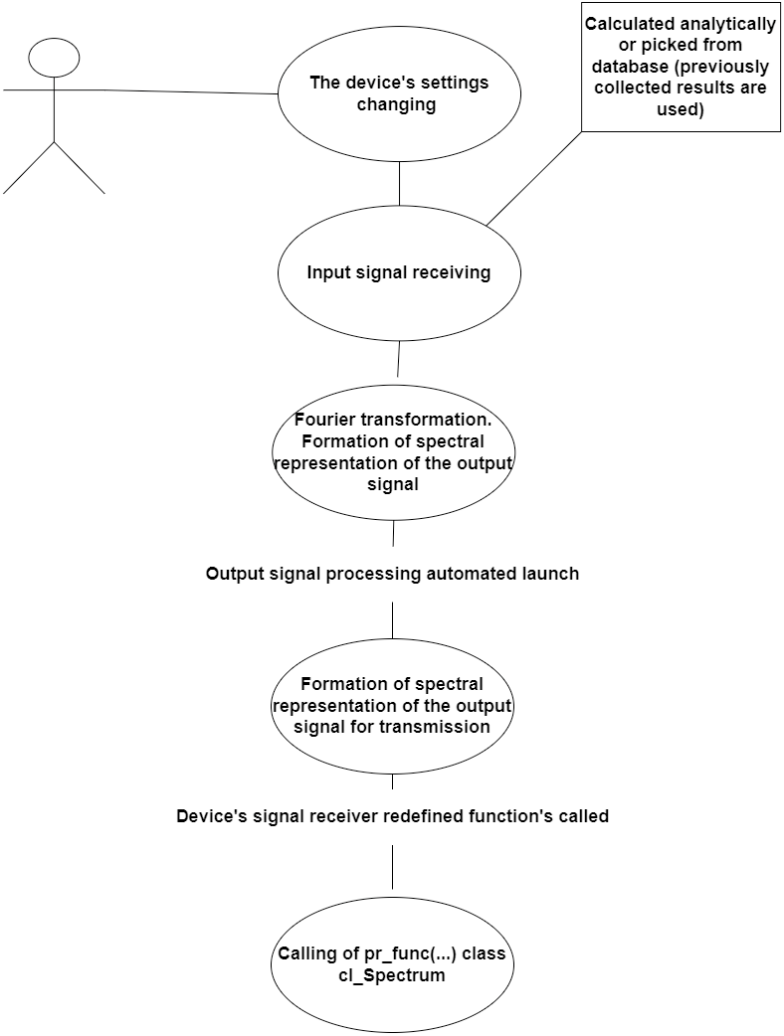
Interaction of the objects during the data transmission.

Thus, data exchange between devices occurs at the initiative of the studied device. When the parameters of the input radio signal are changed (for example, the user has changed the frequency or amplitude of the signal), the software of the device model generates a spectral representation of the output radio signal (arrays: frequency and amplitude) and calls the pr_func() function, through which the data is transferred to the dynamic array of the spectrum analyzer. The interaction between the device being studied and the spectrum analyzer model during data transmission is shown in Fig. 3.

### An example of a virtual lab implementation

**Figure 4:**
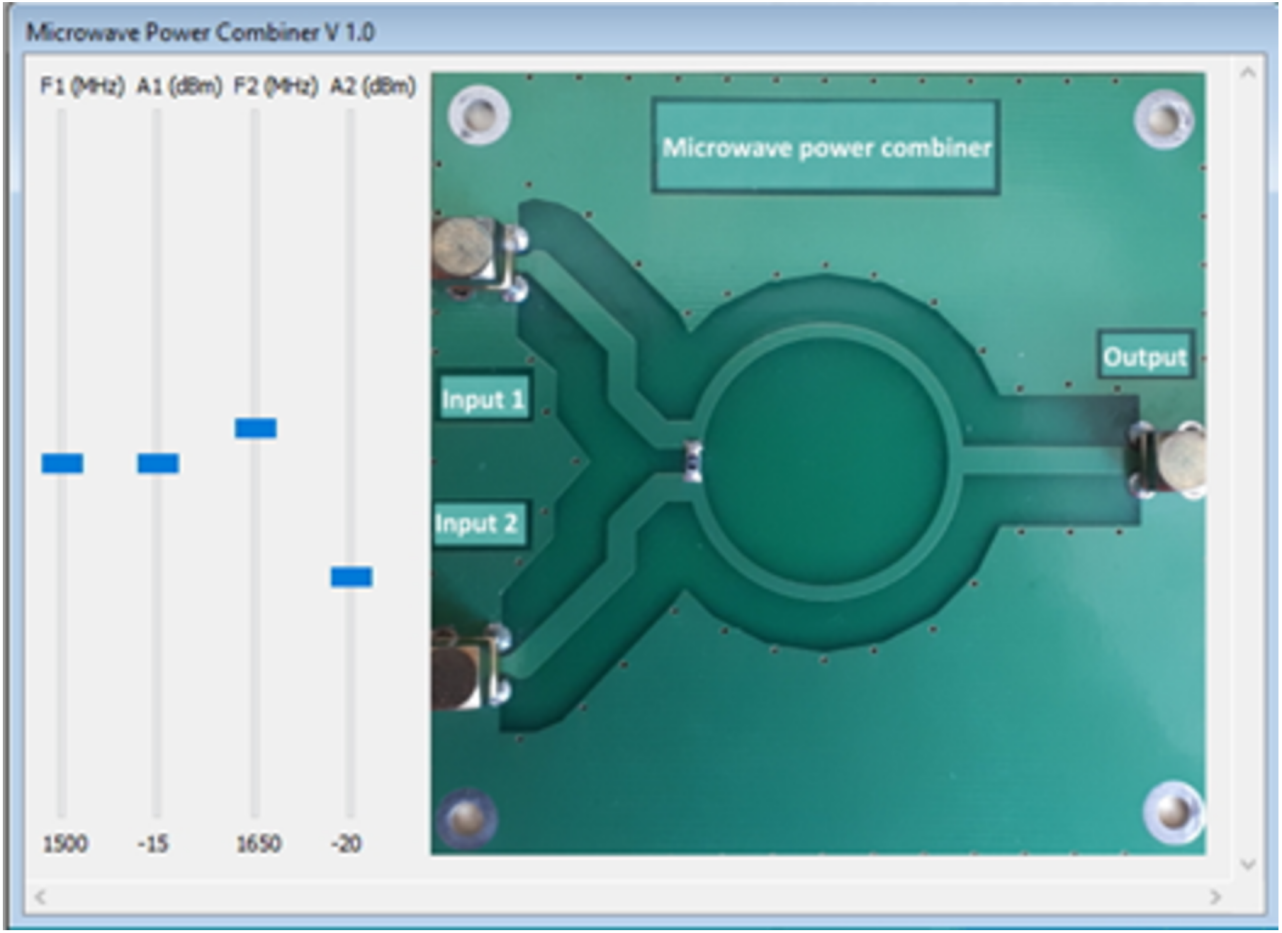
Interaction of the objects during the data transmission.

Let us consider developing a virtual laboratory work that allows the user to gain primary skills in working with a spectrum analyzer. These include the choice of the swath, the installation of markers, and work with the measuring line, in other words—making basic measurements and studying the spectrum of the input radio signal. As the device being studied, we consider a microstrip ring power adder. The computer model of such an adder was created considering the previously stated requirements. Two high-frequency signals are fed to their input, then combined, and, after conversion, fed to the output. In this model, the user can change the frequency of signals from 100 kHz to 3 GHz and their amplitude. Figure 4 shows the computer interface of the created model.

 The microstrip signal adder model can be calculated analytically. For example, let two harmonic oscillations arrive at its input, and then the output signal can be written as:

\begin{eqnarray*}{U}_{out}(t)={U}_{1}\cos \nolimits ({\omega }_{1}t+{\varphi }_{1})+{U}_{2}\cos \nolimits ({\omega }_{2}t+{\varphi }_{2}), \end{eqnarray*}

where *U*_1_ and *U*_2_—input signal amplitudes, *ω*_1,2_ = 2*πf*_1,2_, where *f*_1_ and *f*_2_—input signal frequency, *φ*_1,2_—initial phases, which can be equated to zero.

Further, according to the previously described requirements, a Fourier transform is performed on the resulting output signal in the time domain, and two spectral components are obtained: the first one with frequency *f*_1_ and amplitude *U*_1_, another one with frequency *f*_2_ and *U*_2_ amplitude.

The obtained dataset (an array of frequencies and amplitudes consisting of two elements) is transferred to the input of the spectrum analyzer model for its further processing and visualization on the screen (Fig. 1). The interface of the spectrum analyzer (Fig. 4) allows changing the frequency of input signals in the range from 100 kHz to 3 GHz and their amplitude.

In this lab, device models exchange data in one direction, from the signal combiner to the spectrum analyzer. Therefore, only when the input signals change (*e.g.*, frequency and/or amplitude) it is necessary to recalculate and update the output dataset. The entire data exchange takes place according to the developed concept (Fig. 3)

**Figure 5:**
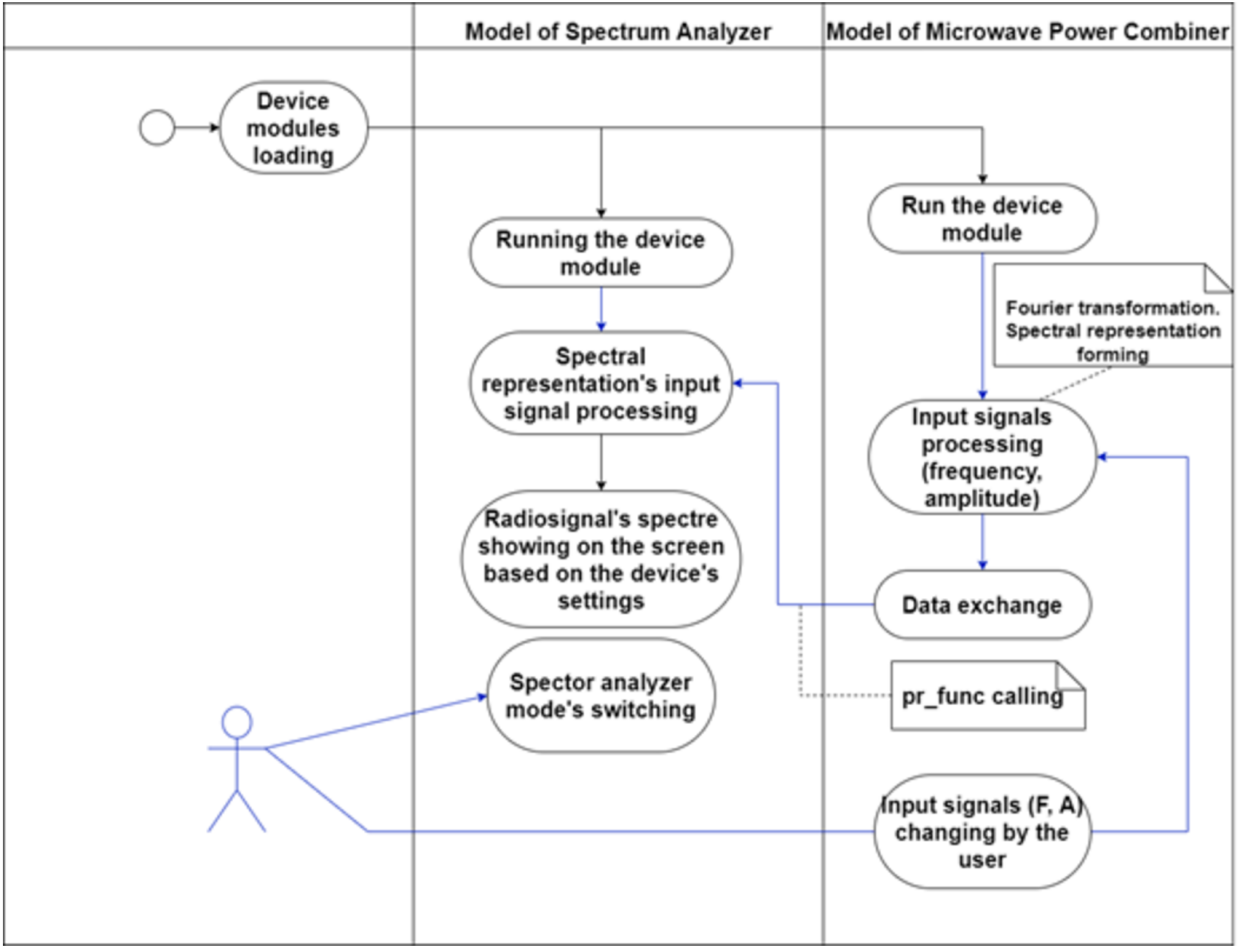
Example of interaction between lab modules.

When the user selects and starts this lab, two dynamically linked libraries are loaded: a spectrum analyzer model and a microwave signal adder model (Fig. 5). At the same time, we consider that, following our concept, the output signal of the power adder is connected to the spectrum analyzer’s input in the virtual laboratory configurator.

This operation is performed when the lab is started, and the user changes the settings for the device under study each time. This made it possible to change the input signals and, in real time, observe the change in their spectral representation on the screen of the analyzer model.

Figure 5 shows that the user can make changes to the mode of operation of the spectrum analyzer, which, at the same time, redraws the spectral representation of the input signal in real time. Thus, a complete feeling of realistic operation on an actual spectrum analyzer is created.

The performance of the developed models was compared to the performance of the actual devices. The results of the same operations were equal, which proves that the virtual models might substitute the actual equipment. The identity of the virtual interface with the real one helps to develop the necessary skills to work with the measurement tools, so the user can set the span, change different parameters, and install markers. In general, it helps to get the appropriate level of skills and experience to interact with the equipment.

It is planned to improve the model of the adder of high-frequency signals in the future. So instead of “scrollbar” changing the frequency of signals, the developed models of high-frequency signal generators (for example, HAMEG Instruments HM8135) can be connected.

### Application in the educational process

This virtual laboratory was implemented and tested in the educational process at the Faculty of Computer Engineering and Cybersecurity of the International Information Technology University (Kazakhstan). Forty-eight students of the *Radio Engineering* academic program participated in the survey. They performed the lab online due to the epidemiological situation in Almaty (Kazakhstan). The work is a part of the *Satellite Communication Systems* course during the fall semester of the 2021-2022 academic year. Before the survey, the guidelines describing the lab goals and procedures were introduced to the students. We developed the questionnaire, which consisted of thirty-seven questions on various application characteristics. It was based on Nielsen’s ten usability heuristics for user interface design (Nielsen, 1994). This approach helps to evaluate different features of the application along with the general user’s satisfaction. All the sections of the questionnaire cover the evaluation of the user interface, the usability of the application, the evaluation of practical use, and the aspects that were liked the most and the least. Also, the test contained questions to check honesty, which allowed us to determine whether a student got acquainted with the application. In addition, there were open questions that helped get users’ feedback. The structure of the survey was as follows: 32 questions with answers in the form of a five-point scale, where 1 is “completely disagree”, 2 is “rather disagree”, 3 is “neutral”, 4 is “rather agree” and 5 is “completely agree”; 3 questions to test the honesty of a respondent with the answer options *Yes* and *No*; and 2 open questions to identify sections that were not reflected in the general questions according to the user. The questionnaire was conducted on Google Forms. Table 2 demonstrates the results of the questionnaire.

**Table 2:** The results of the survey on the virtual lab integration.

Questions	Percentage of positive answers given	Percentage of neutral answers given	Percentage of negative answers given
*The application helps to absorb the material better*	75	18.75	6.25
*After using the application, it is easier to understand the material*	97.9	0	1
*I understood the information studied better after using the application*	93.75	2.08	4.16
*I would recommend the application to friends/acquaintances*	89.5	6.25	4.16

Table 3 includes the standard deviation of the questionnaire results computed using MS Excel, excluding 5 questions. Questions 11–13 have two option answers: “YES” and “NO”, and Q36–37 were open questions on the students’ suggestions on the app’s improvement. Based on the calculations following conclusions can be drawn. Since standard deviation shows the fluctuation of the values from average, our values approve the common positive perception of the virtual lab as it is close to zero. In terms of the answers and appropriate description of the point scale, almost all students found the application sufficient, useful, and necessary for the educational process.

**Table 3:** The average and standard deviation of the questionnaire results. Includes the standard deviation of the questionnaire results computed using MS Excel, excluding 5 questions. Questions 11–13 have two option answers: “YES” and “NO”, and Q36–37 were open questions on the students’ suggestions on the app’s improvement. Based on the calculations following conclusions can be drawn. Since standard deviation shows the fluctuation of the values from average, our values approve the common positive perception of the virtual lab as it is close to zero.

Question number	Average value	Standard deviation
1.	4.3	0.9
2.	4.31	0.9
3.	4.4	0.9
4.	4	1
5.	4.1	0.9
6.	4.3	0.8
7.	4.3	0.8
8.	4.29	0.77
9.	4.4	0.9
10.	4.4	0.9
11.	4.2	1
12.	4.4	0.8
13.	4	1
14.	4.5	0.6
15.	4	1
16.	4	1
17.	4.5	0.6
18.	4.5	0.7
19.	4.3	0.9
20.	4.3	0.9
21.	4.6	0.5
22.	4.3	0.9
23.	4.6	0.7
24.	4.7	0.5
25.	4.1	0.9
26.	4.3	0.9
27.	4.4	0.7
28.	4.56	0.65
29.	4.75	0.434
30.	4.104	1.092
31.	3.92	1.14
32.	4.33	0.93

Based on the obtained results, a high assessment of the practical benefits of the application can be distinguished. Thus, for example, the answers to the questions “*The application helps to better absorb the material*”, “*After using the application, it is easier to understand the material*”, and “*I understood the information studied better after using the application*” have an average of 4.5 points.

From the data obtained, it can be concluded that most students find using virtual labs effective in studying the subject, improving the perception of information, and making it more straightforward. In addition, the students note the application’s usefulness and that it meets the majority’s expectations.

The effectiveness of laboratory work is also reflected in the student’s response to the question, “I would recommend the application to friends/acquaintances”. Thus, 89% of the respondents answered positively. Also, the application meets all the needs of students during the lab implementation. It allows them to study the necessary material and gain relevant experience. Therefore, the application meets the needs of 75% of students.

## Discussion

This article describes implementation process of the model of the spectrum analyzer and virtual lab on the study of the summation of radio signals. Also, the introduction process of the lab work into the educational process and its results are presented. The authors conducted research on the affection of the virtual labs on the quality of the educational process. So first the work observes the design, research, and implementation process of a virtual laboratory’s computer model of a spectrum analyzer. The pandemic has sped up the transfer to distance learning and has helped investigate digital tools’ influence on the educational process. However, the transition has shown all the weaknesses of educational systems and has demonstrated that there were no tools for practical learning, which are especially important for engineering specialties. The previous work (Gubsky et al., 2021) showed the general description of the development of virtual labs in terms of radio engineering, electronics, and telecommunications. This article gives a detailed explanation of the inner construction of the laboratory work and the interaction between its devices. The article describes in detail the interaction of the spectrum analyzer and the device being studied, which is the source of the radio signal. The process of data exchange between them is considered. The article briefly considers the calculation of a microstrip signal adder, the model of which is used as the studied device.

Further, the article describes the process of using the created laboratory work in the educational process. The experience of the students after introducing the new approach in the Faculty of Computer Engineering and Cybersecurity at the International Information Technology University (Kazakhstan) is presented. Students have experienced the work with a virtual model of a spectrum analyzer and were able to work with it remotely. This approach answers to the needs of the active learning process and, based on the survey results, improves students learning experience by encouraging their independence and curiosity. The study results show that the students are highly interested in the proposed method. It provides them with real-world experience since computer models and virtual environments suit the actual equipment and its behaviour. This approves that such simulators are relevant not only in engineering specialties but in every area where practical experience is more important. This approach is relevant in medicine, for example (Georgieva-Tsaneva & Serbezova, 2020). The outcomes of the article confirm the previous hypothesis of the researchers since users’ experience and satisfaction highlighted in the survey results approve that learning outcomes of the virtual laboratory work enrich students’ practical knowledge and positively affect the educational process. Even in distance learning, students can interact with realistic models of different devices, which means they can start the work with a real one (Sus et al., 2020).

A fair analysis of the proposed approach requires observation from all sides. Therefore, the assignment must help distinguish all the advantages and flaws. Using virtually simulated models provides students with relevant experience and the opportunity to work under real-life conditions, not depending on the device’s location, time, or quantity. Moreover, the computer model has all the necessary features obligatory for students. Also, students have greater freedom as it’s impossible to break anything within the simulation, and they are not afraid of doing something wrong.

On the other hand, this gamification approach makes interaction with the equipment too secure. It makes this virtual experience playful rather than educational. It is a complementary tool rather than a full substitution for the real equipment (Allcoat et al., 2021). Despite the target functionality, each modeled device must have an error mode. It makes the experience closer to the real one and forces students to take a more responsible approach during work. Another problem is that high-level realistic virtual simulations sometimes may require devices with higher technical features than students and end-users have. Also, interaction within a virtual environment must have proper instruction describing what needs to complete within the activity.

## Conclusions

The article reflects work on developing computer models of instruments and devices. Specifically, it describes a model of the spectrum analyzer HMS 3000 from Rohde & Schwarz (Munich, Germany) and its integration into the measurement laboratory. The computer model of the measuring device is identical to the actual device in terms of the user interface. The model’s design and later integration into the educational process within the virtual laboratory work during the global pandemic allow research to test how students evaluate it and how it influences their perception and cognition. Results of the survey conducted among participants of the test launch approved its efficiency and enriched user experience. It allowed students to interact with actual equipment in a remote mode. However, the approach has some flaws, such as a lack of error mode and sometimes redundant gamification that affects the educational experience as non-serious. Future work on this project includes implementing new devices to integrate them into a range of accessible equipment. This will expand the functionality of the virtual laboratory and help students to obtain new competencies and knowledge. There is also a need for an error mode to bring virtual models closer to realistic conditions and make the user experience even more live.

##  Supplemental Information

10.7717/peerj-cs.1130/supp-1 Supplemental Information 1 Results of the questionaire The answers of our respondents after use of the application.

10.7717/peerj-cs.1130/supp-2 Supplemental Information 2 Code for Analyzer

## Additional Information and Declarations

### Data Availability

The following information was supplied regarding data availability: The raw data and the source code of the spectrum analyzer are available in the Supplemental Files.
